# Supplementary material for: Impact of temperature on Downs herring (Clupea harengus) embryonic stages: First insights from an experimental approach
Source: PLoS One. 2023 Apr 7;18(4):e0284125. doi: 10.1371/journal.pone.0284125 (PMC10081806; doi:10.1371/journal.pone.0284125)

Figure S1: Temperature recorded from a Marel Carnot buoy (code 6200443) in front of Boulogne-sur-Mer between mid-November 2021 and mid-January 2022 (Coriolis data; Lefebvre, 2015).

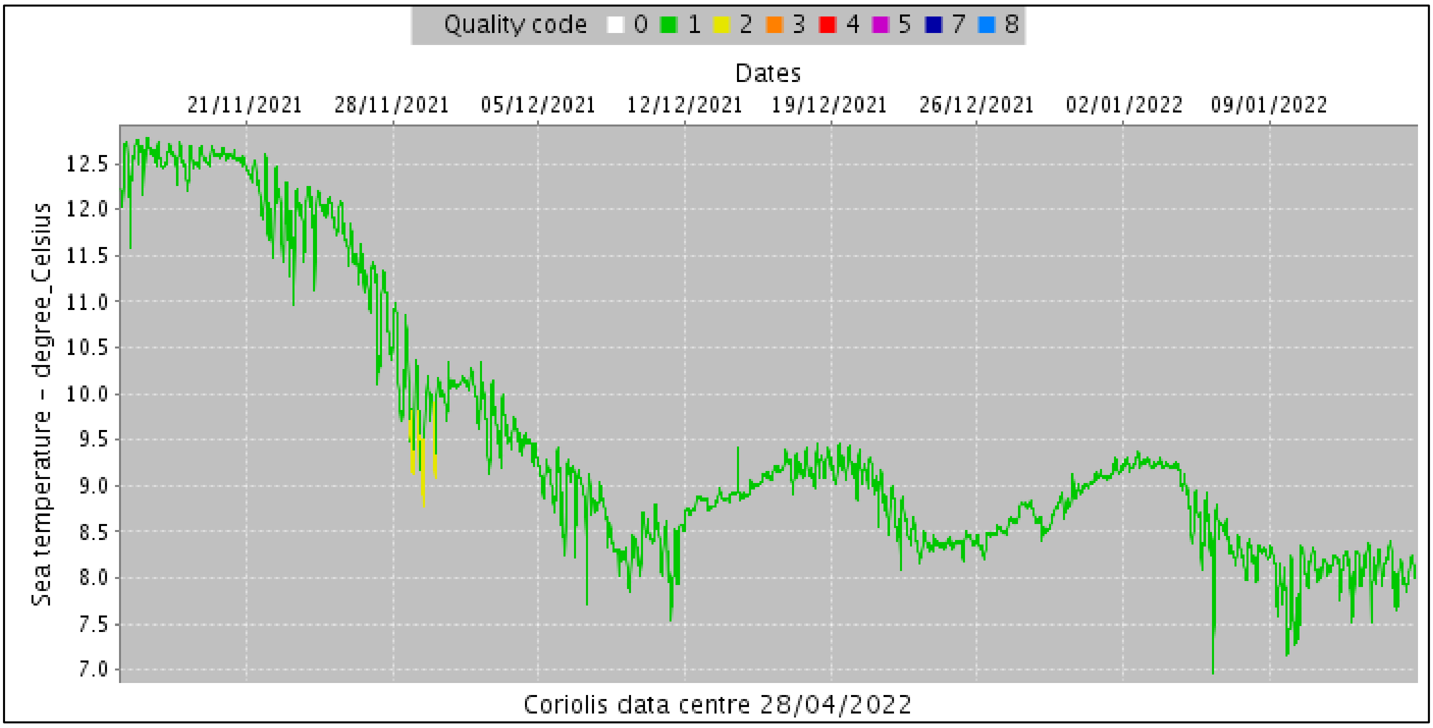

Supplement: S1 Fig — (PDF) [file pone.0284125.s001.pdf]
