# Supplementary figures and images for: Impact of temperature on Downs herring (Clupea harengus) embryonic stages: First insights from an experimental approach
Source: PLoS One. 2023 Apr 7;18(4):e0284125. doi: 10.1371/journal.pone.0284125 (PMC10081806; doi:10.1371/journal.pone.0284125)

**Figure S2:** Experimental set-up

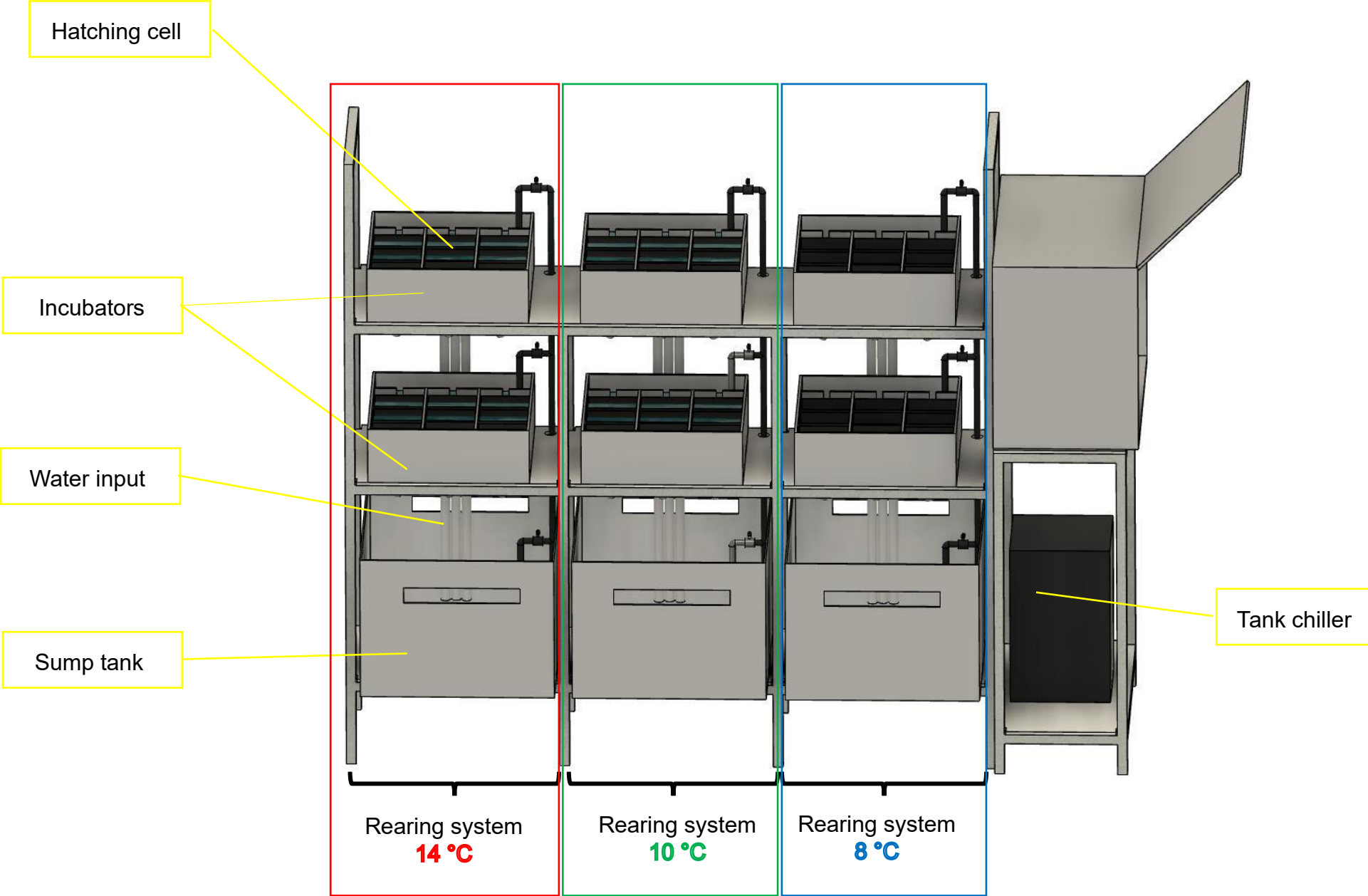

Supplement: S2 Fig — (PDF) [file pone.0284125.s002.pdf]
