# Supplementary material for: Impact of temperature on Downs herring (Clupea harengus) embryonic stages: First insights from an experimental approach
Source: PLoS One. 2023 Apr 7;18(4):e0284125. doi: 10.1371/journal.pone.0284125 (PMC10081806; doi:10.1371/journal.pone.0284125)

Figure S3: Morphological characteristics from pools of males (n=3 per pool) used for the fertilization of eggs from the different females.

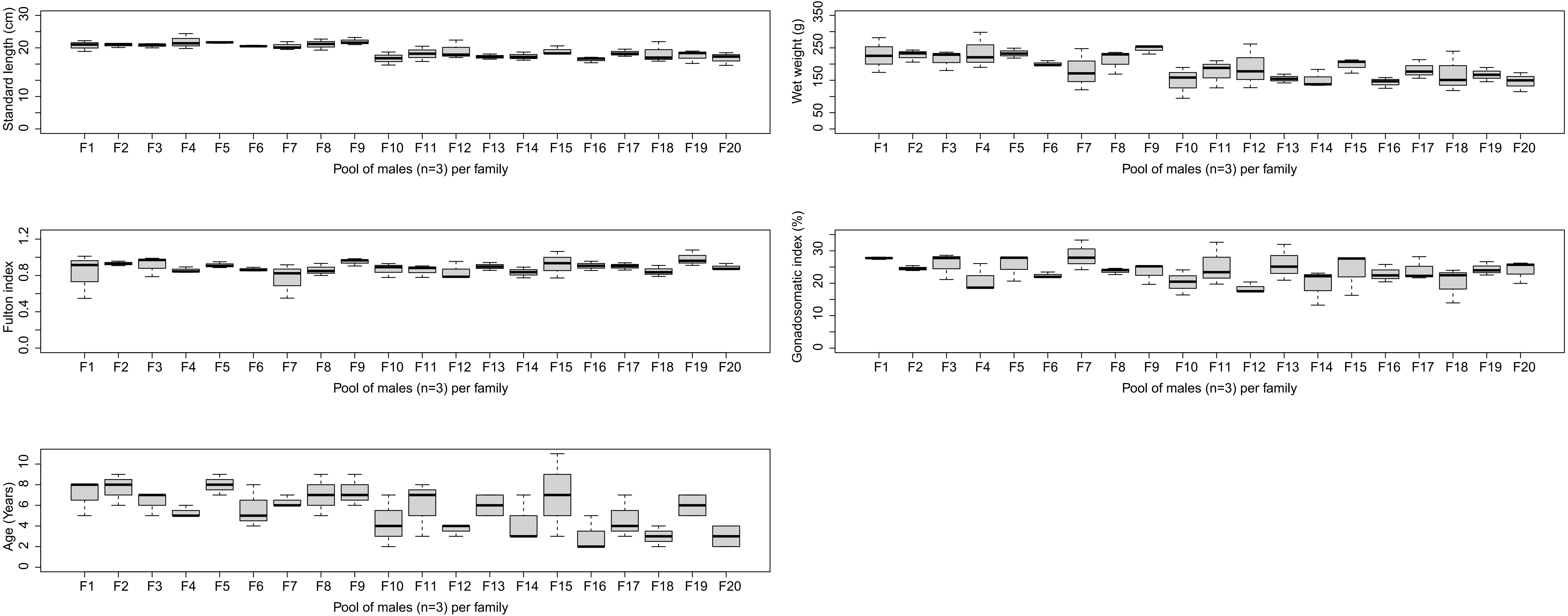

Supplement: S3 Fig — (PDF) [file pone.0284125.s003.pdf]
