# Supplementary material for: Impact of temperature on Downs herring (Clupea harengus) embryonic stages: First insights from an experimental approach
Source: PLoS One. 2023 Apr 7;18(4):e0284125. doi: 10.1371/journal.pone.0284125 (PMC10081806; doi:10.1371/journal.pone.0284125)

Figure S4: Boxplots of fertilization rate across the different families. Colors correspond to the three temperature scenarios: 8 °C (blue), 10 °C (green) and 14 °C (red).

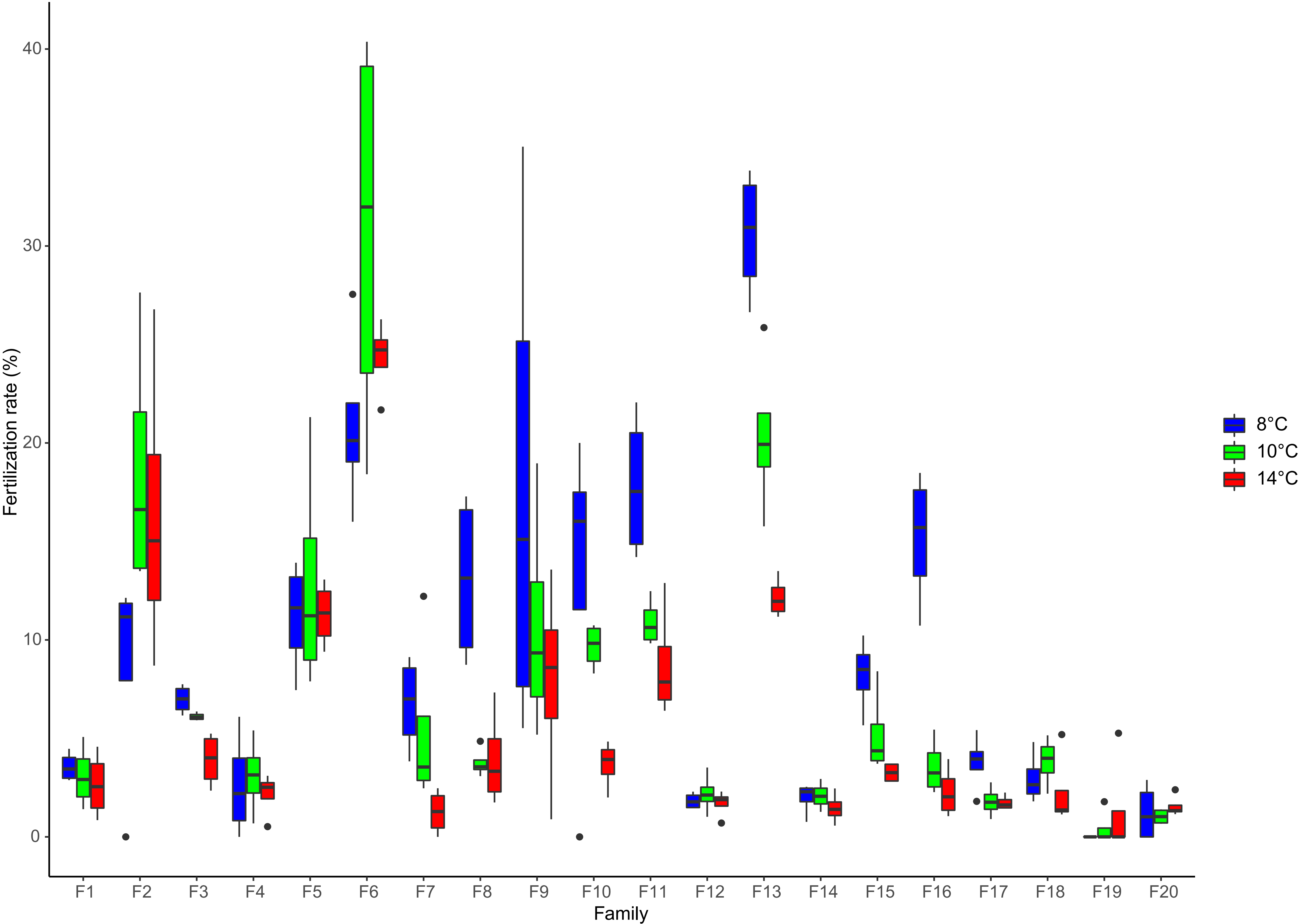

Supplement: S4 Fig — Colors correspond to the three temperature scenarios: 8°C (blue), 10°C (green) and 14°C (red). (PDF) [file pone.0284125.s004.pdf]
