# Supplementary material for: Impact of temperature on Downs herring (Clupea harengus) embryonic stages: First insights from an experimental approach
Source: PLoS One. 2023 Apr 7;18(4):e0284125. doi: 10.1371/journal.pone.0284125 (PMC10081806; doi:10.1371/journal.pone.0284125)

Figure S5: Correlation plot of female attributes. Positive correlations are indicated in red while negative correlations are indicated in blue. Color intensity and circle size are proportional to correlation coefficients.

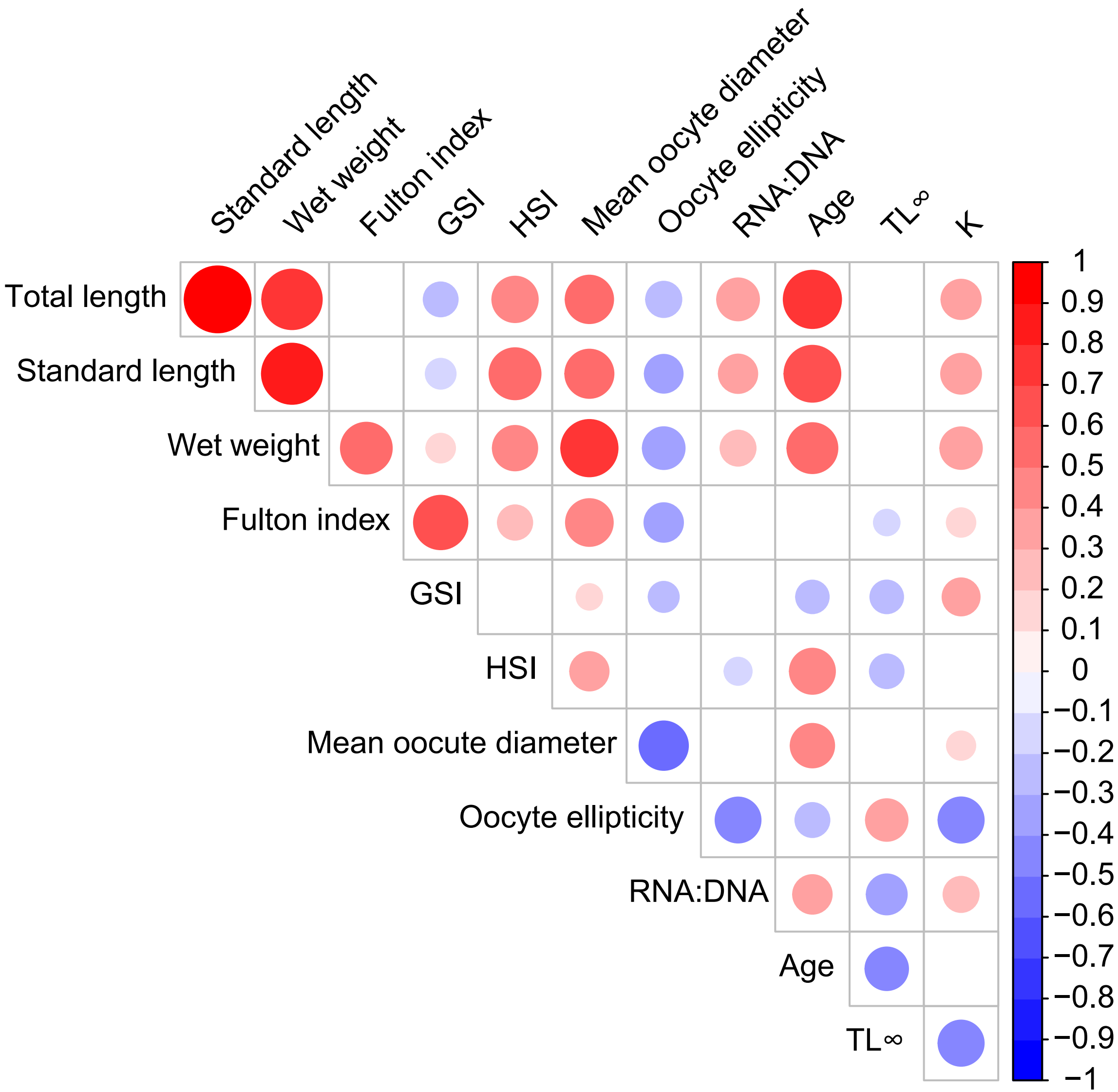

Supplement: S5 Fig — Positive correlations are indicated in red while negative correlations are indicated in blue. Color intensity and circle size are proportional to correlation coefficients. (PDF) [file pone.0284125.s005.pdf]
